# Regional variations in the morphology of the efferent ductules: a histological perspective in rats, bulls, stallions, and boars

**DOI:** 10.1186/s12917-025-05137-5

**Published:** 2025-11-24

**Authors:** Renner Philipe Rodrigues Carvalho, Kamilla Dias Paes Silva, Camilo Jose Ramirez-Lopez, Luiz Otávio Guimarães-Ervilha, Thaina Iasbik Lima, Carlos Mattos Teixeira-Soares, Arabela Guedes de Azevedo Viana, Iara Magalhães Ribeiro, Monica Morais-Santos, Jose Carlos Montes Vergara, Mariana Machado-Neves

**Affiliations:** 1 Postgraduate Program in Veterinary Medicine, Department of Veterinary, Federal University of Viçosa, Viçosa, Brazil; 2 Laboratory of Structural Biology, Department of General Biology, Federal University of Viçosa, Av. P.H. Rolfs, S/N, DBG, ECS333, Campus Universitário, Viçosa, 36570-900 Brazil; 3 Department of Animal Biology, Federal University of Viçosa, Viçosa, Brazil; 4 Department of Animal Science, Universidad de Córdoba, Monteria, Colombia

**Keywords:** Fertility, Sperm maturation, Epididymis, Epithelia, Histomorphology

## Abstract

**Background:**

Infertility is a global concern in both humans and domestic animals, with male factors accounting for nearly half of all cases. Among male reproductive structures, the efferent ductules play a crucial but understudied role in fertility. These tubules connect the testis to the epididymis and are primarily responsible for luminal fluid reabsorption, a process essential for sperm concentration, maturation, and transport. This study aimed to provide a comparative morphological and histological characterization of the efferent ductules in four species—rats, bulls, stallions, and boars—focusing on regional variations (proximal, middle, and distal) and their potential functional implications.

**Results:**

Gross dissection and histological evaluation revealed a columnar epithelium composed of ciliated and nonciliated cells in all species. Comparative analysis identified species-specific differences in connective tissue composition, smooth muscle thickness, and epithelial dimensions. Proximal regions generally exhibited larger lumen diameters and lower epithelial heights, whereas distal regions showed increased ciliary height and, in some species, a greater presence of cytoplasmic granules, suggesting enhanced secretory or absorptive activity prior to sperm entry into the epididymis. Boars exhibited a particularly thick smooth muscle layer and abundant granules in the distal region. Morphometric measurements supported these observations, demonstrating consistent patterns of structural variation across species.

**Conclusions:**

The efferent ductules display complex, species-specific architecture and specialized regional features that likely contribute to their functional role in fluid reabsorption and sperm modification. These findings expand current knowledge of male reproductive morphology in domestic animals and highlight the need for further functional studies beyond rodent models to better understand fertility regulation in veterinary species.

## Background

Infertility has been a persistent concern throughout history and remains a critical issue across species, including humans and domestic animals [1–3]. In humans, it is estimated that 48.5 million couples globally are affected by infertility, a trend that continues to rise [4–6]. While comprehensive studies on domestic animals are limited, the available data suggests that subfertility is increasing, with negative implications for both animal welfare and farm economics [7]. Infertility results from a combination of male and female factors, but male factor infertility contributes to nearly half of all cases [8]. This issue is particularly critical in the context of farm animals, where semen from a single male can be used to inseminate thousands of females. Infertile males can significantly impact conception rates, leading to substantial economic losses for farmers [7].

Sperm dysfunctions are widely recognized as a leading cause of male infertility [9, 10]. For spermatozoa to achieve fertilization capability, they must detach from the seminiferous epithelium and travel through the epididymis - an essential process for their maturation and acquisition of motility [11]. While some sperm dysfunctions stem from defective spermatogenesis [12], others may result from inadequate sperm maturation due to compromised epididymal function [13, 14]. Although research in reproductive biology traditionally emphasizes epididymis for its role in sperm transport and storage, it is equally critical to recognize the pivotal functions of the efferent ductules.

Efferent ductules are a series of small and delicate tubules that connect the testis to the epididymis, emerging separately from the *rete testis*. However, beyond this structural role, these ductules perform critical functions that are essential for male fertility. The principal function of efferent ductules is a luminal fluid reabsorption, that increases testicular fluid concentration by 28 times before they reach the epididymis, ensuring the optimal environment for sperm maturation, concentrating and storage in this duct [15, 16]. The epithelium of the efferent ductules generally consists of two main cell types: ciliated cells, which are responsible for agitating and homogenizing the luminal contents and preventing ductal blockages [17], and nonciliated cells, which play a crucial role in fluid absorption. Fluid reabsorption includes various processes, such as solute transport, passive water permeability, endocytosis and secretion [18, 19]. Significant variation in the structure of the epithelium, as well as in the proportion of these cell types, has been observed along the length of the ductules, leading to their segmentation into three distinct regions: proximal, middle (or conical), and distal [18, 20].

Regional differences in the efferent ductules have been observed not only within individuals of the same species but are even more pronounced across different species [19, 21]. Despite these structural and, consequently, functional variations, most of what is currently known about efferent ductules is based on studies in rodents [22, 23] or humans [24, 25]. This is largely due to their challenging location in larger mammals, as they are often embedded in dense connective tissue occupying part of the epididymis head [19]. In this context, few studies have examined these aspects in farm animals such as bovines [26], equines [27], and swine [28] which leaves a significant gap in our understanding of their reproductive biology and how these structures may influence fertility across species.

In this context, this study aims to provide a detailed analysis of the efferent ductules through macroscopic dissection and histological examination, focusing on the distinct morphological features of each region - proximal, middle, and distal. This approach will deepen the understanding of how the morphology of the efferent ductules is organized within individual species.

## Materials and methods

### Animals and ethics statement

All experimental procedures were reviewed and approved by the Ethics Committee for the Use of Experimental Animals at UFV (CEUA; protocols 1064/2021 and 1076/2021). The selection criteria for sample collection were based on the health and sexual maturity of each species. The minimum ages considered for sexually mature individuals were 76 days for rats [29], 15 months for bulls [30], 24 months for stallions [31], and 7 months for boars [32].

## Rats

Five male adult Wistar rats (70 days old; 200–250 g) were supplied by the Central Animal Facility of the Center of the Biological and Health Sciences of the Universidade Federal de Viçosa (UFV). They were maintained individually in polypropylene cages under controlled photoperiod (12 h light/dark cycle) and temperature (21 °C) with free access to rat chow and water *ad libitum*. This study was carried out in strict accordance with the recommendations of the Guide for the Care and Use of Laboratory Animals (National Research Council, 2011). After 68 days, (138 days old; 350–400 g) the rats were weighed and euthanized by deep anesthesia (ketamine 150 mg kg^− 1^ i.p. and xylazine 10 mg kg^− 1^ i.p.) followed by cardiac puncture. The testes, efferent ductules, and epididymis were removed, and fixation of the dissected efferent ductules was performed by immersion in 10% formalin buffered with PBS (pH 7.4) for 24 h.

## Bulls

Reproductive organs (testes, efferent ductules, and epididymis) were collected from five crossbred bulls (over 30 months of age) sourced from a commercial slaughterhouse located in Muriaé (21° 7’ 49’’ S, 42° 22’ 3’’ W), Brazil. The animals were not specifically slaughtered for this study; rather, samples were obtained opportunistically from animals already destined for commercial meat production. Collection was performed with authorization from the slaughterhouse management. The testes, efferent ductules, and epididymis were fixed using the testicular peripheral perfusion technique, followed by total immersion. For this, two bottles, each connected to a macro drip set, were suspended approximately 1.5 m above the bench to ensure a good flow of solution during perfusion. One bottle contained 9% saline solution with 1.000 IU of heparin per liter, and the other, 10% buffered formalin with PBS (pH 7.4). Using a 22G catheter, the largest peripheral testicular vein was cannulated to begin perfusion with the saline solution to clean the blood vessels and facilitate the perfusion of the fixative through the organ. The flow was maintained for about 10 min until clear fluid exited through the pampiniform plexus. Then, perfusion of the fixative solution (10% buffered formalin with PBS, pH 7.4) was initiated and continued for approximately 20 min. Once perfusion was completed, the sample was reduced via transverse sectioning of the dorsal testicular pole and the epididymal caput, ensuring that the efferent ductules remained attached to a small portion of the testis and the epididymal caput to preserve the anatomical orientation. The sample was then immersed in the fixative solution for 24 h. After this immersion period, the samples were stored in 70% alcohol until they could be fully dissected and processed for histology.

## Stallions

Sample of the reproductive organs (testes, efferent ductules, and epididymis) were obtained from five Mangalarga stallions (aged 5 to 7 years old) during routine clinical-surgical procedures at the Veterinary Hospital of the Universidade Federal de Viçosa, from private veterinarians serving in Viçosa (20° 45′ 14″ S, 42° 52′ 55″ W), Brazil, and surrounding cities, between May and July. The orchiectomy was carried out by the veterinarians on the respective properties, under the supervision of our team. Following the surgical removal, the samples were immediately processed using a protocol similar to that employed for bull specimens.

## Boars

For boars, reproductive organs (testes, efferent ductules, and epididymis) were sourced from five animals of the Piau and Commercial Crossbreed breeds (aged 2 to 5 years old) from the Swine Genetic Improvement sector at the Universidade Federal de Viçosa. These animals underwent routine conventional orchiectomy, which was part of the sector’s regular management activities. The surgical procedure was performed by a veterinarian sector, supervised by our team. Immediately after surgical removal, the organs were processed using the same technique described for bulls and stallions.

### Dissection and segmentation of efferent ductules

In rats, the dissection was performed immediately after collection, following the methodology described by Heuser et al. [33]. Briefly, the epididymal caudal ligament was incised using a scalpel blade, allowing the epididymal corpus to be separated from the testis. The fat pad surrounding the epididymal caput was then carefully removed using dissection forceps and a stereoscopic magnifying glass. While one forceps held the pampiniform plexus to stabilize the sample, the other was used to remove the adipose tissue. A stereoscopic magnifying glass was used when necessary to help delineate and identify the efferent ductules. Once the dissection was completed, the efferent ductules were subdivided into three regions: proximal, middle, and distal. In the proximal region, part of the testis was retained to maintain the connection between the efferent ductules and the *rete testis*, preserving the anatomical orientation. Similarly, the distal region was sectioned, keeping part of the initial segment of the epididymis intact.

In bulls, stallions, and boars, the dissection of the efferent ductules was performed using blunt-tipped dissection forceps to remove the adipose tissue and connective tissue capsule. A longitudinal incision was then made with a scalpel through the tunica covering the efferent ductules and the epididymal caput, which was completely removed using the avulsion technique. Fine-tipped dissection forceps and a stereoscopic magnifying glass were then used to remove the connective and adipose tissues surrounding the efferent ductules, fully exposing them. As with rats, the efferent ductules were segmented into three regions - proximal, middle, and distal - to facilitate the evaluation of the entire length of the ductules.

### Histological analyses

Samples from the proximal, middle, and distal regions were dehydrated in crescent series of ethanol (70%, 80%, 90%, 95%, and absolute). Subsequently, these fragments were immersed in a glycol methacrylate solution (Historesin^®^, Leica Microsystems, Nussloch, Germany) for two hours for pre-infiltration, followed by a transfer to a fresh glycol methacrylate solution for infiltration, where they remained for 12 h. The fragments were then embedded by adding a catalyst (benzoyl peroxide) to the glycol methacrylate solution. Transverse sections, 3 μm thick, were obtained using a glass knife on a rotary microtome (RM2155, Leica Biosystems, Nussloch, Germany). Every tenth section was selected for slide preparation. The histological sections were stained with toluidine blue and subsequently mounted between a slide and coverslip using mounting resin (Entellan^®^).

### Morphometric analyses and cell proportion in efferent ductules

Digital images of the efferent ductules were captured using a photomicroscope (Olympus BX53, Tokyo, Japan) and analyzed with Image-Pro Plus 4.5 software (Media Cybernetics, Silver Spring, MD). Each histological section contained several efferent ductule profiles. In each region (proximal, middle, and distal), 30 well-oriented transverse ductule cross-sections per animal were selected for measurements of ductal diameter, luminal diameter, epithelial height, ciliary height, and the relative distribution of epithelial cell types. The raw cell numbers were corrected using Amann’s formula [34].

### Statistical analysis

All quantitative results had their normality evaluated by the Shapiro-Wilk test. Data were submitted to One-way ANOVA followed by the post hoc Tukey test. Differences were considered significant when *p* < 0.05. Statistical analyses were performed using GraphPad Prism (version 6.0, Graph Pad Software Inc., San Diego, CA, USA). Results were expressed as means ± standard deviation (SD).

## Results and discussion

### Characterization of efferent ductules in rats

**Fig. 1:**
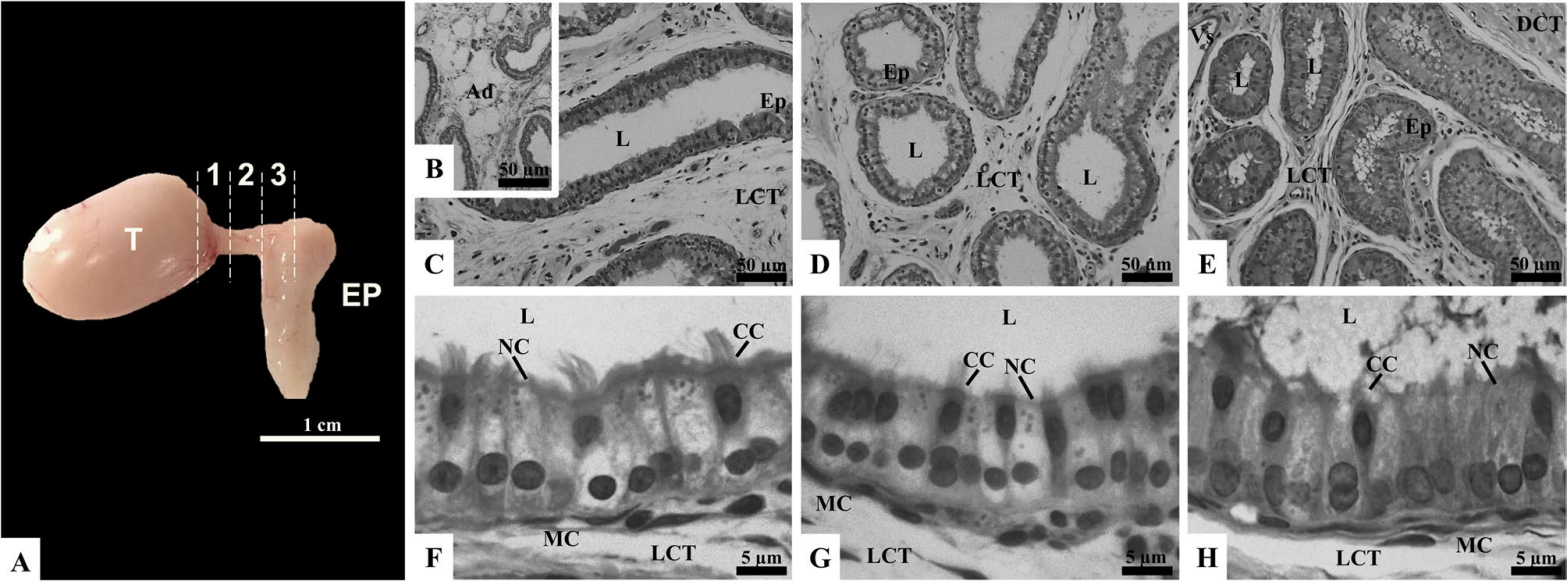
Gross anatomy (**A**) and microscopy (**B-H**) of rat efferent ductules (ED). The ED, situated between the testis (T) and the epididymis (EP), are divided into proximal (1), middle (2), and distal (3) regions. Histological sections of the proximal (B, C, F), middle (D, G), and distal (E, H) regions display adipocytes (Ad), loose connective tissue (LCT), lumen (L), and epithelium (Ep), together with dense connective tissue (DCT), blood vessels (Vs), and muscle cells (MC). The epithelium comprises ciliated cells (CC) and nonciliated cells (NC). Sections stained with toluidine blue and analyzed under light microscopy (*n* = 5 rats)

After incising the epididymal caudal ligament, the most part of loose connective and adipose tissues located in the proximal region of the epididymis covering the efferent ductules and the epididymal caput were carefully removed (Fig. 1A). Macroscopically, the adipose tissue was distinguishable from the efferent ductules by its shiny, whitish, translucent, and friable appearance, while the ductules exhibited an opaque, yellowish and firm consistency. Under the stereoscope, using transillumination, these characteristics were clearly identified, aiding the dissection process. Once the dissection was completed, the conical region in the middle was identified, along with the presence of small blood vessels superficial to the efferent ductules. The ease of applying this technique can be attributed to the size of the organ, which was only surrounded by a small adipose tissue pad. The main caution during dissection was avoiding compression or cutting of the ductules to prevent structural damage, which was effectively managed using fine-tipped forceps, the stereoscope and gently dissection. These characteristics have also been reported by Hess [19], and La et al. [35], who described that rats have between 2 and 8 proximal efferent ductules.

In the histological analysis, unilocular adipocytes were observed around the efferent ductules, primarily located outside the connective tissue of the capsule, but also penetrating the connective tissue internally, as shown in Fig. 1B The presence of these adipocytes suggests a metabolically active environment around the ductules. Adipose tissue may provide a local energy source and be involved in hormone production and other factors that could influence ductule function. For example, it is well established that epididymal fat plays a crucial role in spermatogenesis, maintaining local metabolic and immunological niches [36]. While the impact of fat on testicular physiology is widely recognized, its role in epididymal and efferent ductule physiology remains poorly understood. Additionally, the interstitial space between the efferent ductules presented loose connective tissue, clusters of adipose cells, and blood vessels, highlighting the importance of adequate structural and nutritional support for the ductules. Epithelial secretions and projections from the connective tissue capsule were also observed (Fig. 1C, D, and E).

Throughout their length, the efferent ductules displayed a pseudostratified columnar epithelium composed of ciliated and nonciliated cells, resting on a thin layer of muscle cells (Fig. 1F, G, and H). The rat epithelium also presents some points of depression, resembling a groove, which penetrates almost to the basal membrane, especially in the proximal and middle regions (Fig. 1C and D). The nonciliated cells exhibited a basal ovoid or spherical nucleus and clear cytoplasm with small vacuoles, while the ciliated cells had an apical-central oval nucleus and were equipped with cilia. The presence of these two cell types suggests functional specialization for the absorption and movement of fluid secreted by the germinal epithelium. Nonciliated cells, which contain the majority of endocytic vesicles and vacuoles, are responsible for absorbing fluid from the testis. On the other hand, the ciliated cells likely assist in fluid and sperm movement and homogenization along the efferent ductules [37].

Regarding cell proportions, a predominance of nonciliated cells was observed in both regions of the efferent ductules. However, there was a gradual increase in the proportion of ciliated cells along the regions, with the highest proportion observed in the distal region (*p* < 0.05; Table 1). Other studies confirm that the proximal regions of the ductules have a higher proportion of nonciliated cells, while the distal regions show an increase in the proportion of ciliated cells [38, 39]. This pattern is likely crucial for the transport, absorption, and homogenization of fluid and sperm through ciliary action. It is important to note that the cilia do not move in the same direction; their main function appears to be the agitation of luminal fluids [40]. Additionally, granules were more intensely observed in the proximal region compared to the other regions evaluated (Fig. 1F, G, and H).

**Table 1:** Morphometric parameters and proportion of epithelial cells in the efferent ductules of Wistar rats

Parameters	Region		
	Proximal	Middle	Distal
*Morphometry of efferent ductules (µm)*			
Ductal diameter	207.4 ± 13.9^a^	157.2 ± 27.9^b^	89.2 ± 14.9^c^
Lumen diameter	191.2 ± 12.7^a^	125.4 ± 27.2^b^	47.4 ± 14.9^c^
Epithelial height	16.1 ± 7.9^a^	31.8 ± 1.9^b^	41.9 ± 1.6^c^
Ciliary height	4.99 ± 0.5^a^	8.1 ± 1.2^b^	12.8 ± 0.8^c^
*Proportion of cells (%)*			
Nonciliated cells	86.3 ± 3.0^a^	82.0 ± 2.8^ab^	78.9 ± 4.3^b^
Ciliated cells	13.7 ± 3.0^a^	17.9 ± 2.8^ab^	21.0 ± 4.3^b^

Mean ± SD. Different superscript letters (^a, b, c^) in the same row indicate significant differences between groups (*p* < 0.05) according to Tukey’s post hoc test (*n* = 5 rats)

In the proximal region, the efferent ductules exhibited a larger ductal and lumen diameter, along with a lower epithelial and ciliary height compared to the middle and distal regions (*p* < 0.05; Table 1). Similarly, in the middle region, these parameters showed statistically similar results to those in the distal region (*p* < 0.05; Table 1). In rodents, it is common for the proximal efferent ductules to be straight and have a wider lumen than those found in the distal regions closer to the epididymis [19]. This is particularly relevant considering that testicular fluid production in rats is estimated to be between 30 and 50 µl h^− 1^, with the flow rate reduced to approximately 2 µl h^− 1^ in the fluid entering the epididymis [41]. Therefore, a significant portion of the testicular fluid is reabsorbed in the efferent ductules as sperm transit through the ducts. Most of this reabsorption occurs in the proximal regions, shortly after the fluid exits the *rete testis* [19].

These observations explain the regional variations found, suggesting functional adaptation along the efferent ductules. For example, the larger luminal diameter and higher proportion of nonciliated cells in the proximal regions provide a greater surface area for testicular fluid reabsorption. Additionally, the increased height of the cilia and the higher proportion of ciliated cells in the distal regions indicate a role in homogenizing the fluid as sperm concentration increases. This function may be crucial for the consistent reabsorption of fluid at the lumen/nonciliated cell interface, where up to 90% of the fluid is processed through the epithelium and either metabolized, recycled, or reabsorbed into the epididymal vasculature in a short period [15].

### Characterization of efferent ductules in bulls

**Fig. 2:**
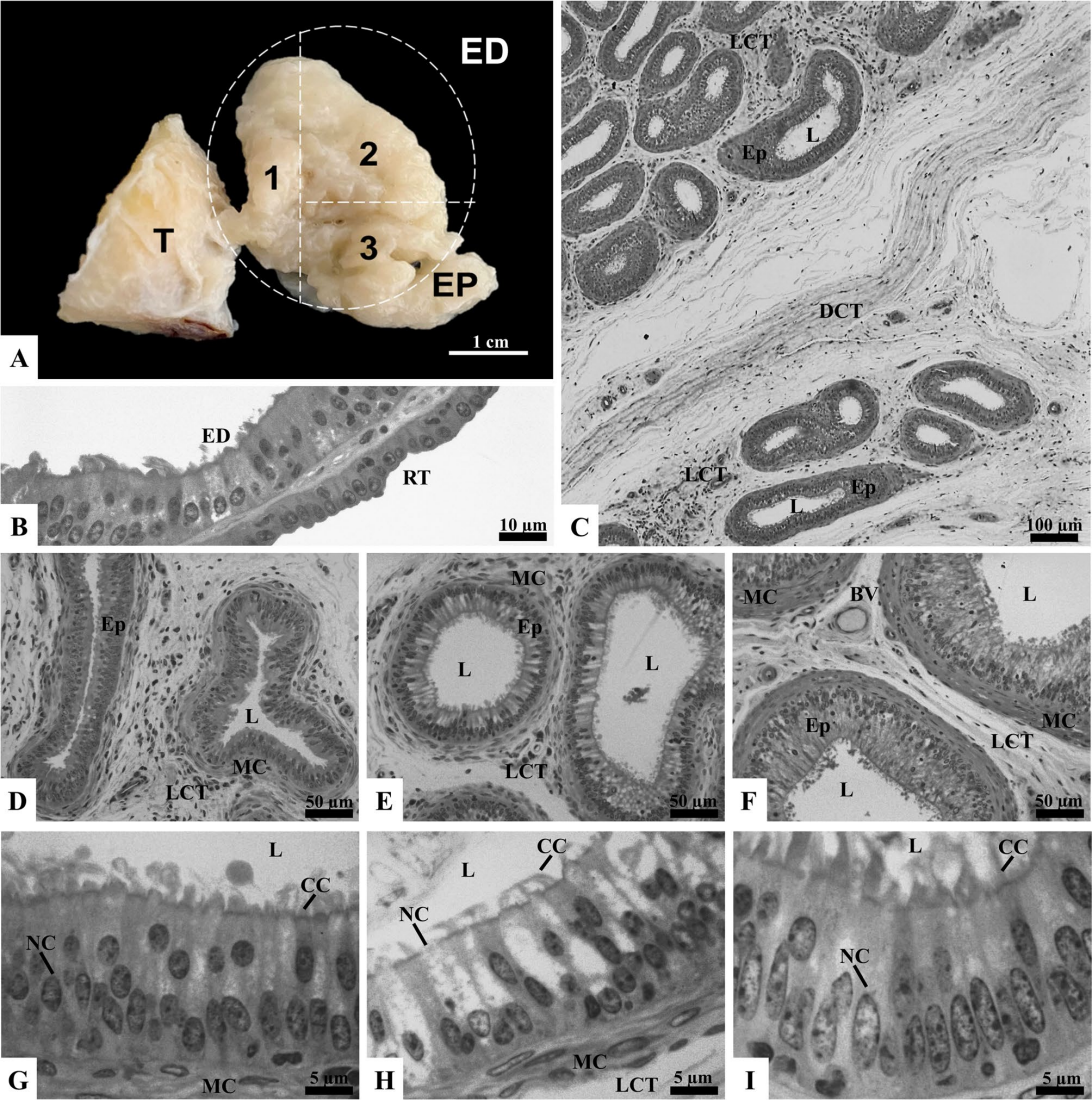
Gross anatomy (**A**) and microscopy (**B**–**I**) of bull efferent ductules (ED). The ED, outlined by the circle, are located between the testis (T) and the epididymis (EP), and are subdivided macroscopically into proximal (1), middle (2), and distal (3) regions. Histological sections include the rete testis (RT), lined by simple cuboidal epithelium, adjacent to the proximal ED, which are lined by pseudostratified columnar ciliated epithelium (B). Bundles of ED surrounded by dense connective tissue (DCT) are shown in panel C. Sections of the proximal (D, G), middle (E, H), and distal (F, I) regions display ciliated cells (CC), nonciliated cells (NC), muscle cells (MC), loose connective tissue (LCT), blood vessels (BV), lumen (L), and epithelium (Ep). Sections stained with toluidine blue and analyzed under light microscopy (*n* = 5 bulls)

In bulls, the removal of the tunica albuginea, identification and dissection of the efferent ductules proved extremely difficult due to the strong adherence of these structures. The ductules exhibited a firm consistency and whitish appearance, which made individual dissection of the efferent ductules impossible by manual means. Therefore, the efferent ductules were subdivided into three sections (Fig. 2A), and the proximal, middle, and distal portions were identified only through microscopic observation. Unlike in rats, histological sections showed that the efferent ductules in bulls were grouped into bundles by dense connective tissue (Fig. 2C), while the rest of the interstitial space consisted of loose connective tissue containing blood vessels (Fig. 2D, E, and F). We hypothesize that the difficulty in dissection is due to the interstitial composition observed in the ductules of these bulls. This fibrous aspect, noted during dissection, has not been described in existing literature, likely because many studies employ chemical microdissection techniques. However, the interstitial characteristics reported in this study are consistent with the analysis of Hemeida et al. [20], who also identified the presence of dense connective tissue surrounding the ductules in bulls, which typically range from 13 to 16 efferent ductules.

Similar to rats, the bull’s efferent ductules throughout their length exhibited a pseudostratified columnar epithelium composed of nonciliated and ciliated cells. This epithelium rests on a thin layer of smooth muscle cells, supported by bundles of loose connective tissue (Fig. 2G, H, and I). The presence of these two cell types suggests functional specialization for absorption, secretion, and movement of fluids and sperm. As reported by Alkafafy and Sinowatz [42], the wall of the bull ductules is supported by a thin lamina propria and a fine layer of smooth muscle cells, and their lumens contain few sperm. Additionally, the thickness and number of smooth muscle cells increase toward the epididymis. Similar findings were reported in the same species by Wrobel [43], where the peritubular interstitial connective tissue is described as rich in connective tissue fibers and contains relatively frequent mast cells.

In the proximal region, upon passing through the tunica albuginea, the extra-testicular *rete testis* gives rise to the efferent ductules, as illustrated in Fig. 2B. This transition is marked by a change from the simple cuboidal epithelium of the *rete testis* to the pseudostratified columnar ciliated epithelium of the efferent ductules. The epithelial transitions between the efferent ductules and the *rete testis*, as well as between the ductules and the epididymis, are described as abrupt in various species, with gradual transitions observed only in humans. Additionally, the *rete testis* epithelium can be simple squamous or cuboidal, depending on the species [44]. The shift from simple cuboidal to pseudostratified columnar ciliated epithelium highlights an adaptation zone where sperm begins their transport through the efferent ductules. This structural change is critical for the transport and modification of seminal fluid in the ductules. The *rete testis* epithelium is known for its high endocytic activity, absorbing and degrading various proteins derived from the seminiferous tubules’ lumen via lysosomes. Some of these proteins are secretory products of Sertoli cells, which may interact with sperm during their formation in the testis but have limited functional importance as they move toward the epididymis. The *rete testis* also seems to function as a cleaning station for some of these testicular-derived proteins. Therefore, while passage through the *rete testis* may seem passive, it plays important roles in fine-tuning the lumen environment for sperm as they enter the efferent ductules [19].

In the proximal region, morphometric measurements revealed smaller ductal and luminal diameters, as well as lower epithelial and ciliary heights, compared to the middle and distal regions (*p* < 0.05; Table 2). Similarly, in the middle region, these parameters were smaller than those in the distal region (*p* < 0.05; Table 2). No descriptive studies were found that address the morphometric particularities of the efferent ductules in bulls. However, this pattern, together with the moderate spermatogenic efficiency of bulls (ten to 20 million sperm per gram of testis per day) [45], may help explain the gradual increase in ductal and luminal diameters toward the distal region. Humans, who also exhibit moderate to low sperm production (less than ten million sperm per gram of testis per day), show a comparable morphological pattern, with the proximal ductules narrower than the distal ones and an increase in diameter toward the epididymis [46]. While this interpretation remains speculative, the contrasting spermatogenic efficiencies among species—higher in boars and stallions and lower in bulls and humans [45]— may underlie the opposite morphological trends observed.

**Table 2:** Morphometric parameters and proportion of epithelial cells in the efferent ductules of crossbred bulls

Parameters	Region		
	Proximal	Middle	Distal
*Morphometry of efferent ductules (µm)*			
Ductal diameter	105.3 ± 21.5^a^	203.7 ± 24.3^b^	300.3 ± 20.5^c^
Lumen diameter	75.9 ± 21.7^a^	169.1 ± 24.8^b^	260.4 ± 20.8^c^
Epithelial height	29.5 ± 1.9^a^	34.6 ± 1.1^b^	39.9 ± 0.9^c^
Ciliary height	6.7 ± 0.2^a^	8.7 ± 0.6^b^	10.9 ± 1.4^c^
*Proportion of cells (%)*			
Nonciliated cells	77.1 ± 3.0^a^	65.7 ± 2.8^a^	66.1 ± 4.3^a^
Ciliated cells	22.9 ± 3.9^a^	34.3 ± 5.5^a^	33.9 ± 7.2^a^

Mean ± SD. Different superscript letters (^a, b, c^) in the same row indicate significant differences between groups (*p* < 0.05) according to Tukey’s post hoc test (*n* = 5 bulls)

Regarding cell types, no differences were observed in the proportions of nonciliated and ciliated cells between the regions of the efferent ductules (*p* > 0.05; Table 2). However, granules were observed more intensely in the middle and distal regions, compared to the proximal region (Fig. 2G, H, and I). The variation in the regional pattern suggests that the function of the ductules becomes more complex and active as sperm progress toward the distal regions. The lower epithelial and ciliary height in the proximal region may be related to a reduced need for testicular fluid modification shortly after sperm exit the testis, while the increased height in the middle and distal regions reflects greater transport and modification activity. Nevertheless, the absence of differences in the proportions of nonciliated and ciliated cells between the regions indicates a relatively uniform distribution of these cell types along the ductules, suggesting a consistent basic functionality across all regions. Although these hypotheses are raised based on observations, there is no evidence in the literature to fully support these assumptions. Further studies are needed to establish a clear relationship between the morphometric characteristics and functionalities of the different regions of the efferent ductules in bulls. It is worth noting that most of the knowledge about efferent ductules is derived from studies conducted on laboratory rodents, and there is a lack of comparable information on efferent ductules in large domestic animals such as bovines.

### Characterization of efferent ductules in stallions

**Fig. 3:**
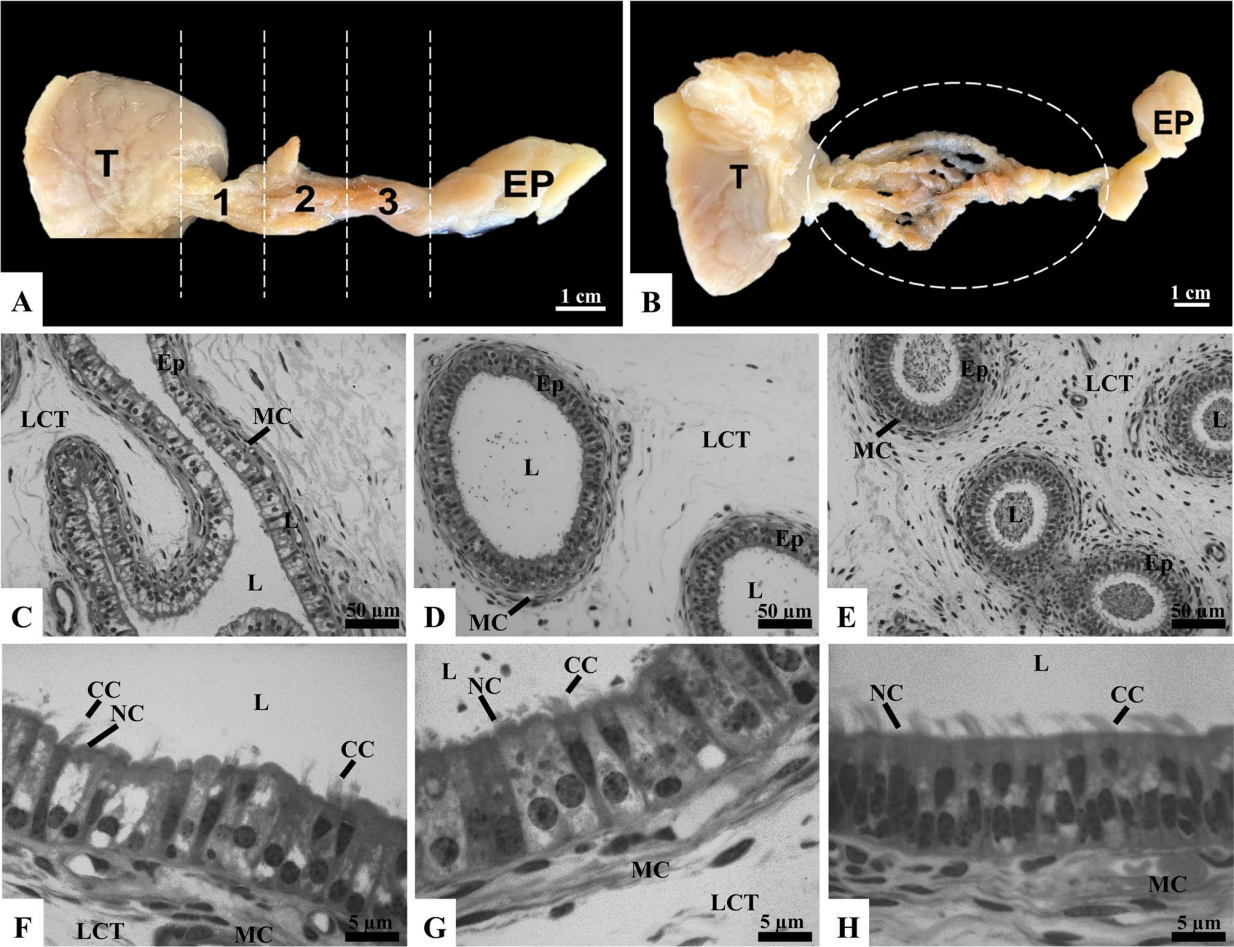
Gross anatomy (**A**, **B**) and microscopy (**C**–**H**) of stallion efferent ductules (ED). The ED, located between the testis (T) and the epididymis (EP), are divided into proximal (1), middle (2), and distal (3) regions. Panel B shows the ED after dissection (dashed circle). Histological sections of the proximal (C, F), middle (D, G), and distal (E, H) regions display the epithelium (Ep), lumen (L), muscle cells (MC), and loose connective tissue (LCT). The Ep comprises ciliated cells (CC) and nonciliated cells (NC). Sections stained with toluidine blue and analyzed under light microscopy (*n* = 5 stallions)

Upon removing the tunica albuginea, the efferent ductules in stallions were predominantly surrounded by loose connective tissue, which facilitated their individualization from the *rete testis* to their insertion into the epididymis, as shown in Fig. 3A and B. Additionally, the efferent ductules exhibited a pinkish-brown coloration and soft consistency, contrasting with the epididymis, which appeared whitish and firm. Amann [47] described in detail that the efferent ductules extend from the extra-testicular rete testis, with each rete tubule fusing with one of approximately 13 to 15 efferent ductules that lead to the epididymal duct. In the proximal caput of the epididymis, the highly coiled distal ends of these ductules merge into a single duct, forming the epididymal duct (Fig. 3B).

Histologically, the efferent ductules were surrounded by thin layers of smooth muscle and loose connective tissue (Fig. 3C, D, and E). In stallions, sperm transport through the epididymal duct primarily occurs via continuous peristaltic contractions of smooth muscle. While the specific role of smooth muscle cells in the efferent ductules remains unclear, studies from other species suggest that the thin layer of smooth muscle may aid in sperm transport. Initially, it was believed that the cilia of the efferent ductules were solely responsible for moving sperm toward the epididymis. However, recent research suggests that smooth muscle contraction assists in sperm movement, while motile cilia create a rotating motion in the testicular fluid, keeping sperm suspended and preventing blockages [17].

The epithelium of the efferent ductules was classified as pseudostratified columnar ciliated. Similar to other species described here (rats and bulls), this epithelium consisted of nonciliated and ciliated cells (Fig. 3F, G, and H). Table 3 presents the results of morphometric parameters and cell type proportions in the stallion efferent ductules. In the proximal region, the ductules showed larger diameters, both in the ductules and the lumen, compared to the middle and distal regions (*p* < 0.05; Table 3). A gradual decrease in these diameters was observed along the middle and distal regions, with the smallest diameters found in the distal region (*p* < 0.05; Table 3). Unlike rats, whose efferent ductules are surrounded by a fat pad, in larger mammals these structures extend through a greater portion of the epididymis, particularly the initial segment [44], which may influence their overall ductal dimensions. No significant differences were found in epithelial and ciliary height between the proximal, middle, and distal regions (*p* > 0.05; Table 3). This absence of regional differences in epithelial and ciliary height observed in stallions, unlike the other species analyzed, may be related to the period of sample collection, which occurred during short-day photoperiod conditions in Brazil (non-breeding season). Indeed, seasonal variations in testicular activity and Sertoli cell populations have been reported in stallions, with higher activity during the breeding season [48]. Although seasonal changes in the efferent ductules of stallions have not been investigated, studies in other seasonal breeders, such as camels, have shown morphological variations in epithelial height and lumen diameter associated with reproductive activity [49]. Therefore, future studies across different photoperiods are needed to clarify this potential influence.

**Table 3:** Morphometric parameters and proportion of epithelial cells in the efferent ductules of stallions

Parameters	Region		
	Proximal	Middle	Distal
*Morphometry of efferent ductules (µm)*			
Ductal diameter	284.3 ± 31.7^a^	201.9 ± 17.4^b^	147.1 ± 12.4^c^
Lumen diameter	250.0 ± 17.8^a^	163.7 ± 12.3^b^	115.6 ± 9.9^c^
Epithelial height	34.3 ± 6.7^a^	38.2 ± 2.1^a^	31.5 ± 1.9^a^
Ciliary height	6.8 ± 0.4^a^	10.1 ± 1.9^a^	9.6 ± 1.5^a^
*Proportion of cells (%)*			
Nonciliated cells	74.3 ± 3.8^a^	65.0 ± 6.1^b^	65.2 ± 5.7^b^
Ciliated cells	25.7 ± 3.4^a^	34.9 ± 6.1^b^	34.8 ± 5.7^b^

Mean ± SD. Different superscript letters (^a, b, c^) in the same row indicate significant differences between groups (*p* < 0.05) according to Tukey’s post hoc test (*n* = 5 stallions)

Regarding cell proportions, nonciliated cells predominated in all regions, but a higher proportion of ciliated cells was observed in the middle and distal regions compared to the proximal region (*p* < 0.05; Table 3). Additionally, qualitative evaluation revealed variation in the presence of granules, with greater intensity in the proximal region compared to the middle and distal regions (Fig. 3F, G, and H). Overall, the proximal regions had a higher proportion of nonciliated cells, while regions closer to the epididymis showed an increase in the number of ciliated cells. The proportion of ciliated cells in the efferent ductules can vary significantly between species and ductal regions, ranging from 1:2 to 1:5 [21], and in some species, ciliated cells can constitute up to 80% of the ductal epithelium [20]. Although specific studies on the proportion of ciliated cells in stallion efferent ductules are lacking, the higher proportion of these cells in the middle and distal regions suggests a role in homogenizing testicular fluid. This is consistent with the observation that the highest sperm concentration in the distal regions results from the reabsorption of most testicular fluid in the proximal regions.

### Characterization of efferent ductules in boars

**Fig. 4:**
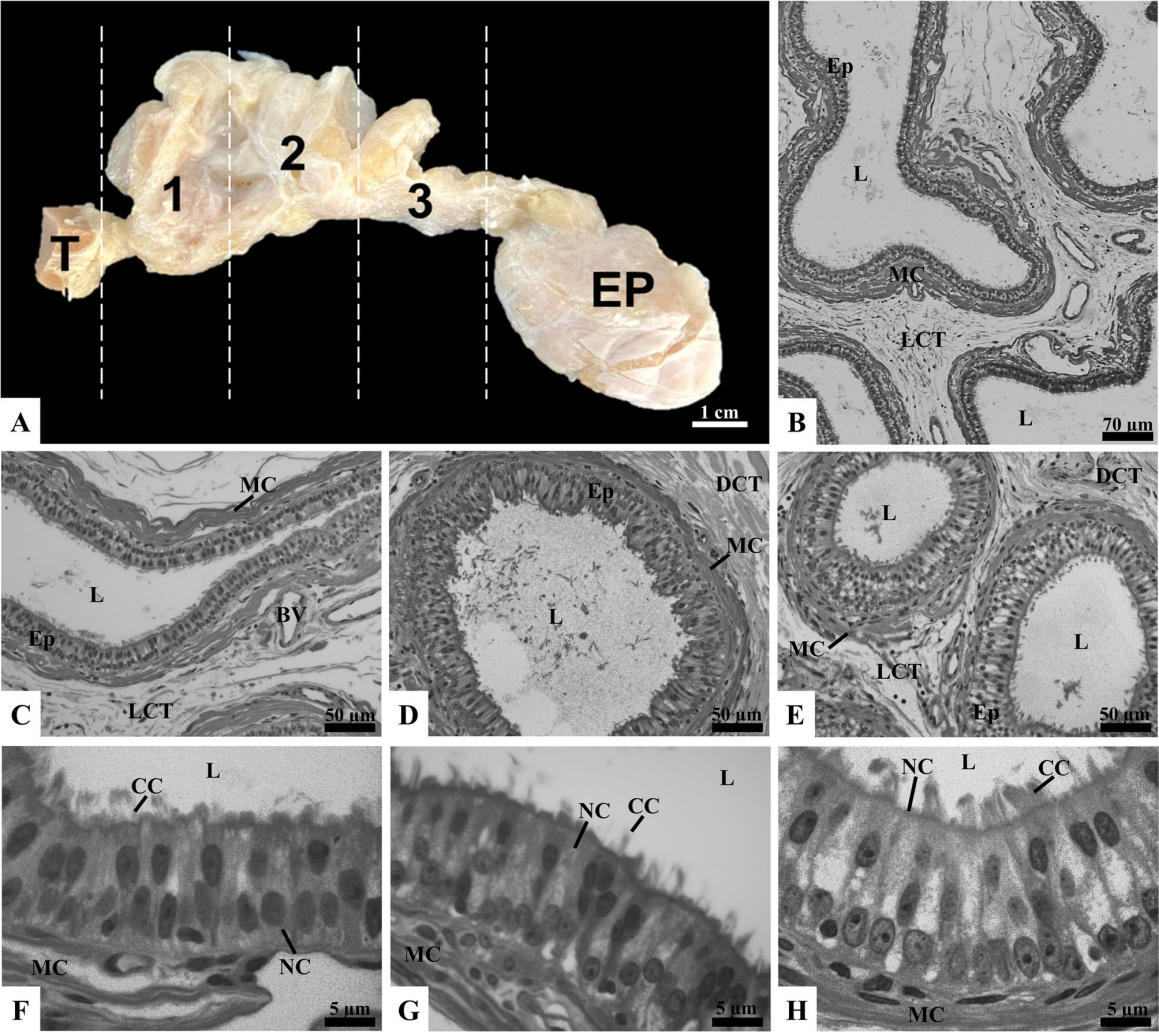
Gross anatomy and microscopy of boar efferent ductules (ED). The ED, located between the testis (T) on the left and the epididymis (EP) on the right, are subdivided into proximal (1), middle (2), and distal (3) regions (A). Panel B shows ED surrounded by a thick muscular layer and connected by loose connective tissue (LCT). Histological sections of the proximal (C, F), middle (D, G), and distal (E, H) regions highlight ciliated cells (CC), nonciliated cells (NC), muscle cells (MC), LCT, dense connective tissue (DCT), lumen (L), and epithelium (Ep). Sections stained with toluidine blue and analyzed under light microscopy (*n* = 5 boars)

After removing the tunica albuginea, the efferent ductules in swine were found to be surrounded by a thick, dense layer of yellowish, opaque, and greasy adipose tissue. The complete removal of this tissue allowed for the identification of the testicular origin and epididymal insertion of the ductules. However, mechanical dissection to individualize the ductules was not feasible due to the fragility of the tissue (Fig. 4A). According to Stoffel and Friess [50], who performed corrosion casts, between 12 and 14 efferent ductules were identified, emerging from the extratesticular anastomosed rete and connecting to the initial segment of the epididymis.

Similar to observations in rats, bulls, and stallions, the efferent ductules in boars displayed a pseudostratified columnar epithelium with cilia, composed of nonciliated and ciliated cells (Fig. 4F, G, and H). Immediately beneath the epithelial tissue, a thick layer of smooth muscle surrounded the ductules, while the interstitial space was predominantly composed of loose connective tissue with numerous scattered blood vessels (Fig. 4B). A notable characteristic of boar efferent ductules is the significantly thicker layer of smooth muscle beneath the epithelial tissue, which is more developed compared to the other species studied (Fig. 4C, D, and E). Given that rhythmic contractions of smooth muscle are crucial for sperm transport through the ductules [17], understanding the physiological aspects that necessitate a thicker muscle layer in swine is essential.

In the proximal and middle regions, the efferent ductules showed a larger diameter compared to the distal region. Additionally, the lumen diameter in all three regions displayed significant statistical differences, with the largest diameter observed in the proximal region, followed by the middle and distal regions (*p* < 0.05; Table 4). Stoffel and Friess [50] reported that the abrupt transition from the cuboidal epithelium of the *rete testis* to the ciliated columnar epithelium of the efferent ductules occurred within the irregularly shaped area of the extratesticular rete. This transition resulted in a wide and irregular lumen, lined either partially or fully by the epithelium of the efferent ductules. Indeed, the proximal segments of the ductules have a larger diameter and a more sinuous course. As seen in rats and stallions, most fluid reabsorption occurs in the proximal regions, immediately after the fluid exits the *rete testis* [43]. Additionally, in the distal regions, the lumen diameter was substantially reduced [51]. The gradual reduction in lumen diameter along the regions may reflect the progressive reabsorption of fluid and concentration of sperm as they approach the epididymis.

**Table 4:** Morphometric parameters and proportion of epithelial cells in the efferent ductules of boars

Parameters	Region		
	Proximal	Middle	Distal
*Morphometry of efferent ductules (µm)*			
Ductal diameter	266.5 ± 14.1^a^	244.9 ± 4.9^a^	207.8 ± 5.3^b^
Lumen diameter	244.6 ± 8.9^a^	197.7 ± 8.7^b^	153.0 ± 6.5^c^
Epithelial height	21.9 ± 9.3^a^	47.2 ± 4.7^b^	54.7 ± 4.5^b^
Ciliary height	7.2 ± 0.7^a^	9.2 ± 0.9^b^	11.5 ± 0.7^c^
*Proportion of cells (%)*			
Nonciliated cells	71.4 ± 3.0^a^	70.4 ± 7.3^a^	65.2 ± 4.5^a^
Ciliated cells	28.7 ± 3.0^a^	29.6 ± 7.3^a^	34.8 ± 4.5^a^

Mean ± SD. Different superscript letters (^a, b, c^) in the same row indicate significant differences between groups (*p* < 0.05) according to Tukey’s post hoc test (*n* = 5 boars)

The epithelial height was lower in the proximal region (*p* < 0.05; Table 4), while no statistical differences were observed between the middle and distal regions (*p* > 0.05; Table 4). Additionally, a gradual increase in ciliary height was observed from the proximal to distal regions (*p* < 0.05; Table 4). This pattern suggests a functional adaptation, where longer cilia in the more distal regions may help facilitate more efficient sperm and fluid movement along the ductules, compensating for the decreased lumen diameter and higher sperm concentration. Regarding cell proportions, nonciliated cells predominated in all regions, but no significant differences were observed between the proportions of nonciliated and ciliated cells across the proximal, middle, and distal regions (*p* > 0.05; Table 4). Granules were observed more intensely in the distal region compared to the other regions (Fig. 4F, G, and H). The higher intensity of granules in the distal region may indicate increased cellular activity in this part of the ductules, contributing to the modification of the luminal environment shortly before the sperm exit the efferent ductules [43].

## Conclusion

This study provided a detailed insight into the morphology and structure of the efferent ductules in rats, bulls, stallions, and boars, highlighting their distinct characteristics and functional specializations. In rats, the dissection was facilitated by the clear distinction between adipose tissue and the efferent ductules, and detailed observation using a stereoscope allowed for precise characterization. Histological findings confirmed that the structure of the efferent ductules is highly specialized, with significant regional variations in the proportion of nonciliated and ciliated cells, reflecting functional adaptations for fluid and sperm absorption and transport. The morphometric characteristics, such as ductal diameter and ciliary height, demonstrate clear adaptation for reabsorption and transport functions, with a higher proportion of ciliated cells in the distal regions, consistent with existing literature. In bulls, the dissection revealed a dense clustering of the ductules, making individualization difficult. However, the histological and morphometric features observed, such as the presence of dense connective tissue and variation in ductal and lumen diameter, align with existing studies, underscoring the complexity of the bovine ductules and the need for further research to understand the specific functions of these variations. In stallions, the presence of loose connective tissue facilitated dissection, and the ductules exhibited a morphological pattern consistent with other species, though with notable differences in ductal diameter and cell proportion along the length of the ducts. The observed pattern of increasing ciliated cell proportions in the distal regions suggests an important role in testicular fluid homogenization and sperm movement. In boars, the thick layer of fat and smooth muscle surrounding the efferent ductules highlights the complexity of these structures in this species. The morphometric differences, such as the larger ductal and lumen diameters in the proximal regions and greater ciliary height in the distal regions, suggest a distinct functional adaptation for reabsorption and sperm transport. The more intense presence of granules in the proximal region may reflect increased cellular activity in this area. Overall, the described characteristics of the efferent ductules reflect specific functional adaptations that allow for the efficient adjustment of testicular fluid transport and modification. These findings contribute to a deeper understanding of the structure and function of the ductules, emphasizing their complexity and importance in reproductive physiology. Future studies should expand knowledge of the functional and structural specifics of the efferent ductules, further contributing to a broader understanding of reproductive physiology in different contexts. Indeed, alterations in the efferent ductules have been linked to significant causes of male subfertility and infertility. However, the mechanisms regulating their activity remain poorly understood. Therefore, further research into microscopic and functional aspects of efferent ductules is essential to advance the field.

## Data Availability

The datasets analysed during the current study are available from the corresponding author on reasonable request.
